# Unmasking the Culprit of Profound Hyponatremia in an Infant

**DOI:** 10.1155/crpe/4883190

**Published:** 2026-01-07

**Authors:** Bayan Matarneh, Richard Johnson, Jamie Bell

**Affiliations:** ^1^ Department of Pediatrics, Children’s Hospital of Michigan, Detroit, Michigan, USA, childrensdmc.org; ^2^ College of Medicine, Central Michigan University, Mount Pleasant, Michigan, USA, cmich.edu

## Abstract

Infants with severe hyponatremia (Na < 120) and an abdominal mass require an astute clinical evaluation and often represent a medical emergency. We report a case of a 2‐month‐old White female, born full term, presenting with a two‐day history of decreased oral intake and urinary output, who was found to have severe hyponatremia with notable abdominal distention. Abdominal imaging revealed the presence of a pelvic mass causing obstructive uropathy. Further imaging and urogenital examination revealed a hydrometrocolpos. This rare entity required urgent surgical intervention and standard supportive care in the PICU. She had a subsequent significant improvement in symptoms and prevention of further deleterious complications.

## 1. Introduction

Abdominal distention in infants often presents a distressing challenge in the emergency room, as etiologies range from benign to malignant, with few distinguishing signs on examination. Timely diagnosis and management are crucial to prevent complications. An unusual cause of infantile abdominal distention is hydrometrocolpos—a rare condition characterized by fluid accumulation in the vagina (hydrocolpos) and/or uterus (hydrometra), typically due to distal vaginal obstruction [1]. This condition can lead to the formation of a compressive mass, resulting in complications such as hydroureteronephrosis, electrolyte imbalance, urinary tract infections (UTIs), and increased risk of sepsis and end‐organ damage. A high level of suspicion is required in this patient population to enable earlier diagnosis and prompt surgical intervention to prevent serious complications [2].

## 2. Case Presentation

A 2‐month‐old female infant, born full term at 41 weeks, presented to the emergency room with oliguria and decreased oral intake for two days, along with progressive abdominal distention and emesis. The baby was fed only by direct breastfeeding, and the parents denied any other forms of water or fluid supplementation. The family reported no fever, trauma, or antecedent infections. She did have a normal newborn screen after birth.

**Figure 1:**
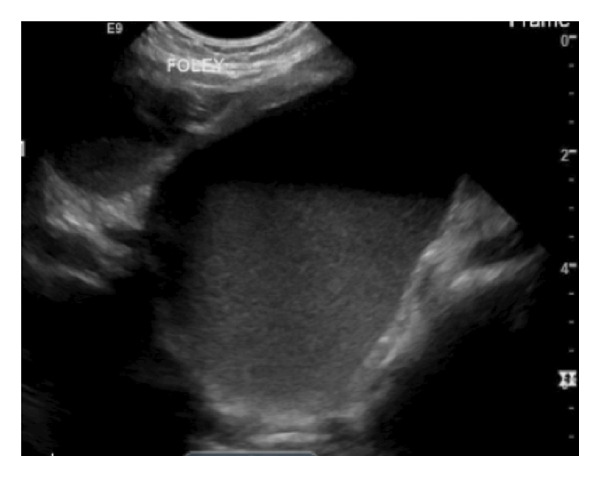
Abdominal US showing bilateral hydroureteronephrosis and a cystic structure in the right hemipelvis.

In the emergency room, vital signs showed a weight of 7.4 kg, temperature of 38.2°C, heart rate of 156 beats/min, respiratory rate of 70 breaths per minute, and blood pressure of 93/56 mmHg. The physical exam showed a tired but alert baby, well‐nourished with no dysmorphic features. The cardiorespiratory exam was significant for tachypnea, mild retractions, and diminished perfusion, with a capillary refill time of > 3 s. The abdomen was soft, diffusely tender with notable abdominal distention, and no organomegaly. She had normal female external genitalia. Laboratory results revealed severe hyponatremia (direct sodium level of 111 mmol/L), hyperkalemia (7.2 mmol/L), acute kidney injury with elevated creatinine (0.5), a blood urea nitrogen (BUN) of 33, and nonanion gap (AG of 13) metabolic acidosis (bicarbonate of 10). Urinalysis tested positive for blood, protein, and leukocyte esterase, and serum WBC was elevated at 43,000, consistent with UTI. Marked abdominal distension prompted an ultrasound, which showed bilateral hydroureteronephrosis and a cystic structure in the right hemipelvis Figure 1.

The infant received two normal saline boluses and one hypertonic saline bolus (5 mL/kg) for resuscitation and then started on maintenance fluids of D5W/0.45% NS and 50 mEq/L sodium acetate to slowly correct her hyponatremia. Blood and urine cultures were sent, and she was given empiric ceftriaxone to treat her UTI. The patient was admitted to the pediatric intensive care unit for further care. A Foley catheter was placed to relieve urinary obstruction, and a subsequent abdominal computed tomography (CT) revealed a large pelvic cystic mass with a suspicious uterine‐like morphology, raising concern for hydrometrocolpos, as shown in Figure 2.

Figure 2Abdominal CT showing the pelvic mass originating from the uterus: (a) coronal section and (b) sagittal section.

**Figure:**
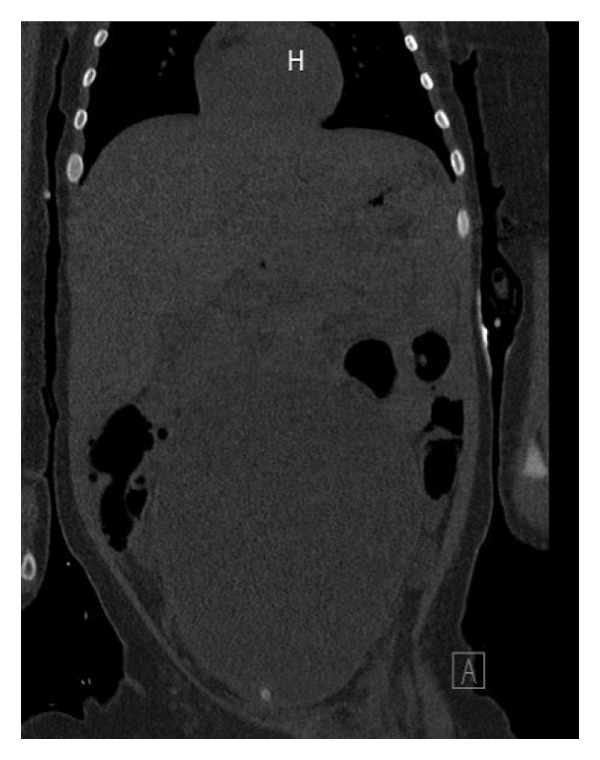
(a)

**Figure:**
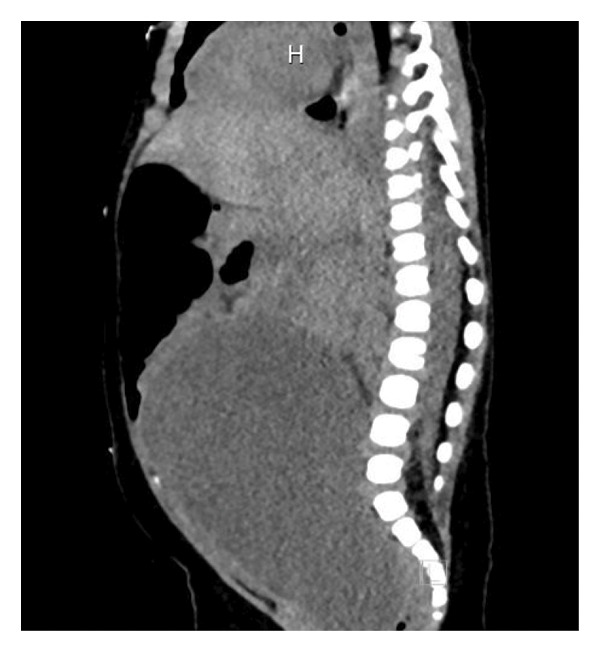
(b)

Urology was consulted, and a cystoscopy was performed. This was followed by an incision and reconstruction of an imperforate hymen, resulting in over 350 cc of thick, milky fluid drainage. A fluid sample was sent for analysis and culture. The patient was continued on ceftriaxone (500 mg) to treat her UTI, and her electrolyte derangements gradually improved over 48 h (see Table 1).

**Table 1:** Electrolytes at presentation and correction over the course of the admission.

Labs	Presentation	After surgery (48 h postpresentation)	On discharge (3 days postpresentation)
Sodium (mmol/L)	111	130	139
Potassium (mmol/L)	7.2	4.1	3.7
Bun/Cr (mg/dL)	33/0.5	23/0.37	9/0.46

Comprehensive urine studies were carried out (Table 2).

**Table 2:** Urine studies.

Test	Value
Urine sodium (mMol/L)	32
Urine potassium (mMol/L)	11.1
Urine chloride (mMol/L)	25
Urine creatinine (mg/dL)	21.5
Urine microalbumin (mg/L)	276
Urine for microalbumin–Cr (mg/dL)	22
Urine microalbumin/Cr ratio (mcg/mg)	1286
Urine osmolality (mOsm/kg)	206
Urine calcium (mMol/L)	1

She was monitored in the ICU for 36 h from admission and then transferred to the acute care floors pending urine culture speciation. Uterine fluid was found to be sterile. Cancer markers were evaluated with no concern for malignancy, including alpha‐fetoprotein (AFP) level 45.4 ng/mL, Cancer Antigen 125 (CA‐125) 31 U/mL, and human chorionic gonadotropin (HCG) 0.9 IU/L (Table 3). The AFP level, though mildly elevated, was within the expected range for a 2‐month‐old child [3].

**Table 3:** Tumor markers.

Test	Value
CA 125	31.2 U/mL
HCG	0.9 IU/L
Alpha fetoprotein	45.4 ng/mL

The patient was ultimately discharged after 3 days with a close urology follow‐up.

## 3. Discussion

A presenting combination of hyponatremia, fatigue, oliguria, and acute kidney injury, in conjunction with abdominal distension/mass, should raise concern for several different pathological processes. This is especially true in the pediatric patient, whose smaller abdominal compartment tolerates space‐occupying processes with much less compliance than that of an adult patient.

The causes of this ominous constellation of symptoms include simple hyponatremic dehydration, sepsis, nephrotic syndrome, endocrinopathies such as congenital adrenal hyperplasia (CAH), intestinal malrotation ± perforation, and intra‐abdominal masses causing mass effect [4]. Therefore, as was done in this case, a thorough evaluation is warranted in such patients. Dehydration and sepsis were present in our patient and were treated promptly with fluids and antibiotics. However, this diagnosis alone was insufficient to explain the presence of her abdominal mass. In our state of Michigan, CAH is tested on the newborn screen, and this patient had normal values as well as no virilization on genital exam, making this diagnosis less likely. Urgent radiographic assessment of the mass noted on the exam was paramount. Given her stability and tolerance of electrolyte derangements, we adopted a stepwise approach, starting with abdominal ultrasonography. Following that, an abdominal CT was pursued to better characterize the abnormalities noted on US (Figure 1).

In our case, a CT abdomen/pelvis scan revealed a large cystic mass of uterine origin within the right hemipelvis, concerning for hydrometrocolpos. This was confirmed during urologic evaluation, leading to diagnostic and therapeutic cystoscopy and incision of the imperforate hymen, the usual standard of care.

One noteworthy aspect of our case is the patient’s age. The typical epidemiological distribution for hydrometrocolpos is bimodal—40% at less than 4 years and 60% at greater than 10 years [5], with a triad of cyclical abdominal pain, amenorrhea, and a negative pregnancy test. The underlying mechanism of hydrometrocolpos is an anatomic obstruction—imperforate hymen, vaginal stenosis, transverse vaginal septum, lower vaginal atresia, or cervical stenosis [6].

The tolerance of such profound hyponatremia, i.e., no evidence of clinical seizures, likely underscores the chronicity of this patient’s pathology. This mass has likely been present and growing since birth/in utero. Mass effect causes progressive obstructive uropathy with backflow of urine through the the ureters and kidneys. Swelling from hydronephrosis damages renal tubules, making them resistant to aldosterone. These patients present with classic lab findings of secondary pseudohypoaldosteronism (PHA): hyponatremia, hyperkalemia, metabolic acidosis, elevated BUN/creatinine, and inappropriately elevated urine sodium levels [7–9], as seen in our patient. In addition, the mass effect of hydrometrocolpos caused urinary stasis and UTI, which likely exacerbated poor feeding and acute dehydration. Her hyponatremia appeared acute on chronic, but with no previous sodium values, it was unclear how much was attributed to her dehydration. For this reason, we attempted to slowly correct her hyponatremia over 36 h to avoid the feared and well‐documented complication of central pontine myelinolysis [10].

It is essential to recognize key differences between CAH and secondary PHA. PHA usually arises when the tubular cells become resistant to aldosterone’s effect, which can be secondary to an obstruction or infection. While CAH is caused by an enzyme defect in cortisol synthesis, which leads to deficient cortisol and aldosterone production. One major differentiating feature is the ambiguous genitalia in CAH, with otherwise normal genital examination in PHA. PHA tends to resolve once we address the obstructive or infectious trigger, while CAH usually requires lifelong treatment [11].

Discharge follow‐up with urology showed a normal urogenital examination, with no recurrence of similar symptoms.

## 4. Conclusion

This unique case of infantile hydrometrocolpos highlights the importance of a thorough physical examination and a broad differential diagnosis in the pediatric setting. Furthermore, prompt abdominal imaging is a key to investigating pediatric abdominal masses. Urgent urological consultation is paramount to help with definitive management in such cases. Her unusual, delayed presentation emphasizes the need for greater access to primary care for our patient population.

## Consent

Informed consent for publication was obtained from the patient’s legal guardian.

## Conflicts of Interest

The authors declare no conflicts of interest.

## Funding

No funding was received for this manuscript.

## Data Availability

The data that support the findings of this study are available from the corresponding author upon reasonable request.
